# *Bacillus Cereus* Catheter Related Bloodstream Infection in a Patient with Acute Lymphoblastic Leukemia

**DOI:** 10.4084/MJHID.2012.004

**Published:** 2012-01-18

**Authors:** N Gurler, L Oksuz, M Muftuoglu, FD Sargin, SK Besisik

**Affiliations:** 1 Istanbul University, Istanbul Medical Faculty, Department of Medical Microbiology, Capa-Istanbul Turkey; 2 Istanbul University, Istanbul Medical Faculty, Department of Haematology, Capa-Istanbul Turkey

## Abstract

*Bacillus cereus* infection is rarely associated with actual infection and for this reason single positive blood culture is usually regarded as contamination . However it may cause a number of infections, such catheter-related bloodstream infections. Significant catheter-related bloodstream infections (CRBSI) caused by *Bacillus spp*. are mainly due to *B. cereus* and have been predominantly reported in immunocompromised hosts. Catheter removal is generally advised for management of infection. In this report, catheter-related bacteremia caused by *B. cereus* in a patient with acute lymphoblast c leukemia (ALL) in Istanbul Medical Faculty was presented.

## Case

A 44-year old man presented with fatigue, weight loss, epistaxis and high fever. Physical examination was insignificant except for pallorness, Complete blood count revealed total white blood cells at 130,000/mm^3^ hemoglobin at 7.6 g/dl, platelet 30,000/mm^3^. He was diagnosed with Pre-B acute lymphoblastic leukemia following bone marrow aspiration, biyopsy and flow cytometric analysis. Cytogenetic analysis showed a karyotype of 45, XY del 9 del 16. The patient achieved complete hematological remission accompanied by disappearance of cytogenetic abnormalities following induction chemotherapy with BFM ALL protocol. The patient was considered to be a candidate for stem cell transplantion because of initial patient characteristics indicating high risk for ALL relapse (Age, complex clonal cytogenetic abnormalities, high total leukocyte count). An HLA-identical unrelated donor, living abroad, was identified. A double-lumen Hickman–catheter (Bard 12.0 Fr, Round Dual Lumen) was inserted by surgical cut-down to access the right subclavian vein which would be necessary for allogeneic stem cell transplantation. Two cycles of consolidation chemotherapy were administered before proceeding to stem cell transplantation. Three weeks later central nervous system (CNS) relapse of ALL occurred, which is treated with four cycles of intrathecal chemoterapy, comprising of methotrexate, cytosine arabinoside and dexamethasone. At the same time a cycle of cytosine arabinoside and cyclophosphamide-based chemotherapy was administered. Stem cell transplantation was planned to be carried out in a month. Three weeks later the patient presented with high fever and headache. Blood cultures were obtained from the catheter and from a peripheral vein and evaluated on the BACTEC 9120 system (Becton Dickinson, USA). *Bacillus spp.* was isolated from the cathether while blood culture obtained from the peripheral vein remained negative. It was regarded as contaminant so that complete identification of *Bacillus* species was not carried out. Lumbar puncture was performed and CNS involvement was documented. Lymphoblastic cells were seen on peripheral blood smear which is considered compatible with hematological relapse. Intrathecal chemotherapy, and methotrexate and high-dose cytosine arabinoside-based systemic reinduction chemotherapy were started. One week after chemotherapy he developed chills and high fever as absolute neutrophil count was 100/mm^3^. The physical examination was noncontributary and blood cultures were collected from the catheter and a peripheral vein. Two blood culture bottles (one from the catheter and one from the peripheral) were positive gram-positive bacilli after three days of incubation at 35°C. The organism produced large, greenish colonies. The bacterium was identified as B.*cereus* on the basis of Gram staining, colony morphology, motility, lecitinase activity. The bacterial identification was confirmed as *B.cereus* using VITEK identification system (bioMeriéux, France). The strain was sensitive to imipenem, vancomycin, gentamicin and ciprofloxacin, whereas resistant to penicillin, cephalosporins and co-trimoxazole. He was started on piperacillin-tazobactam at a dose of 4 g/500 mg every 6 h emprically before blood culture result was available. Vancomycin was added to the treatment regimen due to incomplete clinical and laboratory response. Hickmann catheter, which was inserted about ten months ago, was accepted as the focus of infection and, prolonged maintenance of the catheter was thought to be no more possible. High fever subsided completely upon removal. Hematological remission was achieved and allogeneic stem cell transplantation was scheduled to be performed one week later.

## Conclusion

*Bacillus* species are known to be responsible for several systemic infections, especially in immunocompromised patients. The most commonly reported systemic infection is bacteremia.^2^,^3^ *Bacillus* bacteremia can be serious and even fatal, in immunocompromised patients, such as neutropenia. The most common feature in true *Bacillus* bacteremia is the presence of an intravascular catheter*. B. cereus* produce biofilms, which can play a major role in attachment to catheters.^10^ *Bacillus* species are associated with CRBSIs and they have been well documented, especially among patients with hematological malignancies.^10^^,22^ Some of the reported cases in the literature were shown in the table 1. The table shows that the majority of the patients have abdominal symptoms, but our patients not.

The isolation of *Bacillus* species raises the possibility of contamination on the basis of *Bacillus spp.* being common contaminants of blood cultures.^8^ The ability of *Bacillus spp* to form a biofilm matrix and the adherence properties account for their relation with central venous catheter (CVC) infections.^4^ Catheter-related *Bacillus spp* infections are difficult to eradicate because of slime formation and biofilm production on the catheter surface. Most published data are based on small case series and data regarding the management of *Bacillus spp*. infections are still lacking. Recently conducted study, which is currently the largest series reported, has concluded that retention of the catheter beyond 72 hours after the onset of bacteremia was related to a higher incidence of recurrent *Bacillus* bacteremia.^2^ The drug of choice for *Bacillus* infections is vancomycin.^5^ Despite the proper agent and appropriate length of treatment the clinical outcome remains unsatisfactory. This is largely due to inactivity of vancomycin against the organisms dwelling in biofilm layer and slime production, making the organism highly adherent to the catheters. Based on preliminary data and available “Infectious Diseases Society of America (IDSA)” guideline early catheter removal should be the cornerstone of *Bacillus* bacteremia management.^2^,^6^,^7^ In compatible with this report, our patient developed intermittent fever spikes under the appropriate antimicrobial therapy even though neutropenia resolved with G-CSF support. The Hickmann catheter was removed on the 10^th^ day of vancomycin therapy. Septicemia-related thrombocytopenia unresponsive to aggressive thrombocyte transfusion hindered early removal of the catheter. Catheter tip culture remained negative, which is attributed to ongoing antibiotherapy consisting of piperacillin-tazobactam and vancomycin.

According to the IDSA guidelines published in 2009,^6^ *Bacillus* CRBSIs should be considered after blood contamination is ruled out on the basis of multiple positive culture results, with at least one blood culture sample obtained from a peripheral vein. It is also suggested that the catheters should be removed if the diagnosis is certain, except for in patients having uncomplicated CRBSI involving long-term catheters because of limited access sites and requiring long-term vascular access for survival.

*Bacillus cereus* is a growing concern as a cause of life-threatening infections in patients with hematologic malignancies.^9^ *B.cereus* should be suspected in immunosuppressed patients with intravascular catheter. *B.cereus* septicemia may be fatal in immunocompromised hosts despite broad-spectrum appropriate treatment. Catheter removal is essential for prevention of recurrent bacteremia. Long-term catheter salvage should be reserved for appropriate patient group.

## Figures and Tables

**Table 1:** Reported cases of immunsupressed patients with *Bacillus cereus* bacteremia.

Reference	Year	Age	Diagnosis	Intravenous catheter	Abdominal symptoms	Outcome
11	1988	67	Acute lymphoblastic leukemia		Present	Death
12	1993	43	Acute myelogenous leukemia	Yes	Present	Death
12	1993	15	Acute myeloid leukemia	Yes	Present	Death
13	1997	20	Acute lymphoblastic leukemia(relapse)		Present	Death
14	1997	64	Acute myeloid leukemia	Yes	Present	Death
13	1998	10	Acute lymphoblastic leukemia(relapse)		Present	Recovery
15	1999	13	Acute lymphoblastic leukemia	Yes	Present	Survived
15	1999	15	Acute lymphoblastic leukemia(relapse)	Yes	Present	Death
16	1999	30	Acute myeloid leukemia	Yes	Present	Death
16	1999	43	Acute myeloid leukemia	Yes	Present	Death
16	1999	14	Acute lymphoblastic leukemia	Yes	None	Survived
17	2002	37	Acute myeloid leukemia		Present	Recovery
3	2003	5	Hemophilia	Yes	None	Recovery
18	2005	34	Acute lymphoblastic leukemia	Yes	Present	Death
19	2006	33	Biphenotypic acute leukemia	Yes	Present	Death
20	2006	23	Acute myeloid leukemia	Yes	Present	Death
20	2006	34	Acute lymphoblastic leukemia	Yes	None	Recovery
20	2006	71	Acute myeloid leukemia	Yes	None	Recovery
21	2008	64	Acute myeloid leukemia		Present	Death
**Present case**	**2010**	**44**	**Acute lymphoblastic leukemia**	**Yes**	**None**	**Recovery**
